# A136 EXPLORING ENDOSCOPIST PERCEPTIONS OF ARTIFICIAL INTELLIGENCE-AIDED COLONOSCOPY: A QUALITATIVE ANALYSIS

**DOI:** 10.1093/jcag/gwad061.136

**Published:** 2024-02-14

**Authors:** C Lee, C H Parker, L W Liu, M Salim, T Jeyalingam

**Affiliations:** Toronto Western Hospital, Toronto, ON, Canada; Toronto Western Hospital, Toronto, ON, Canada; Toronto Western Hospital, Toronto, ON, Canada; Toronto Western Hospital, Toronto, ON, Canada; Toronto Western Hospital, Toronto, ON, Canada

## Abstract

**Background:**

Artificial intelligence (AI) is gaining recognition as a promising adjunct in healthcare including gastrointestinal endoscopy. Randomized controlled trials have demonstrated improved polyp detection with computer-aided detection (CADe) systems in colonoscopy. Despite the foreseeable translation of CADe systems into the endoscopy suite, no study to date has explored how introducing this novel technology influences endoscopist perceptions and cognitive processes.

**Aims:**

To explore how AI assistance impacts endoscopists’ perceptions of and cognitive processes during colonoscopy.

**Methods:**

Faculty in gastroenterology and general surgery at the University Health Network (Toronto, Canada) were interviewed in April 2023 to explore their baseline perceptions of AI before the planned installation of the Medtronic GI Genius^TM^ Intelligent Endoscopy Module, an AI polyp detection tool, at Toronto Western Hospital in May 2023. After performing a minimum of 10 colonoscopies using GI Genius^TM^, participants were re-interviewed to discuss their perceptions of AI-assisted colonoscopy and how it influenced their cognition during the procedure. Analysis was informed by constructivist grounded theory, whereby interview data were transcribed and coded iteratively using constant comparison to generate themes.

**Results:**

In this interim constant comparative analysis, 9 participants (6 gastroenterologists and 3 general surgeons) were interviewed to generate 16 interview transcripts (9 pre-exposure and 7 post-exposure; 1 interview pending, 1 participant on leave). Participants held the view that: (1) AI will inevitably become the standard of care in colonoscopy, where clinicians and AI function symbiotically and in partnership; (2) CADe systems may facilitate standardization of practice and reduce inter-endoscopist variability by improving detection of specific types of lesions (e.g., sessile serrated polyps); (3) CADe assistance provides a “second set of eyes” that interacts with, but does not replace, endoscopists’ cognitive processes; and (4) trainees should gain familiarity with AI systems, despite mixed opinions regarding AI’s specific role in training.

**Conclusions:**

We found that study participants conceptualize AI as an essential component of colonoscopy practice and training, with endoscopist thought processes and CADe systems appearing to function symbiotically. Elucidating the specific ways in which AI and endoscopist cognition interact will facilitate the future refinement of these tools.

**Funding Agencies:**

None

